# Rohlin Distance and the Evolution of Influenza A Virus: Weak Attractors and Precursors

**DOI:** 10.1371/journal.pone.0027924

**Published:** 2011-12-06

**Authors:** Raffaella Burioni, Riccardo Scalco, Mario Casartelli

**Affiliations:** 1 Dipartimento di Fisica e Instituto Nazionale si Fisca Nucleare (INFN), Università di Parma, Parma, Italy; 2 Department of Biochemistry, University of Zurich, Zurich, Switzerland; University of Zaragoza, Spain

## Abstract

The evolution of the hemagglutinin amino acids sequences of Influenza A virus is studied by a method based on an informational metrics, originally introduced by Rohlin for partitions in abstract probability spaces. This metrics does not require any previous functional or syntactic knowledge about the sequences and it is sensitive to the correlated variations in the characters disposition. Its efficiency is improved by algorithmic tools, designed to enhance the detection of the novelty and to reduce the noise of useless mutations. We focus on the USA data from 1993/94 to 2010/2011 for A/H3N2 and on USA data from 2006/07 to 2010/2011 for A/H1N1. We show that the clusterization of the distance matrix gives strong evidence to a structure of domains in the sequence space, acting as weak attractors for the evolution, in very good agreement with the epidemiological history of the virus. The structure proves very robust with respect to the variations of the clusterization parameters, and extremely coherent when restricting the observation window. The results suggest an efficient strategy in the vaccine forecast, based on the presence of “precursors” (or “buds”) populating the most recent attractor.

## Introduction

There is a long history in approaching DNA and RNA sequences as texts, with quantitative estimates for various kinds of statistical properties and complexity indicators [1], [2]. The general idea behind this approach is that the information encoded in the sequence is strictly related to the properties of the corresponding biological structures and that good indicators should be able to recognize similar functions in different sequences.

The sector devoted to estimate the relevance of mutations along a time ordered set of evolving sequences is particularly interesting when a sufficiently long record of samples is accessible, as for viral RNA of rapidly evolving diseases [3]–[6]. We focus on a definite kind of statistical properties, precisely metric properties.

The distance between sequences is a concept admitting several implementations. In the context of evolving viral RNA, distances based on the sequence symbols are mostly of the Hamming type: for two strings 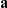 and 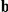 of characters, the Hamming distance 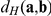 is the number of sites with different symbols [7]–[9]. Such distances are sensitive to local features only, since mutations occurring at different sites are non correlated. In this framework, an interesting solution has been proposed in [8], [9], where, focusing on particular locations of the sequence (the epitopes, whose role and peculiarities in Influenza Virus evolution are well known), authors succeeded in extracting important features of strains evolution. In a sense, the extra information introduced with the choice of the epitopes proved efficient in overcoming the intrinsic uncorrelation of the Hamming metrics, leading to interesting results. Other relevant approaches, based on sequences information only, rely on entropic distances [10]–[14] referred to the Shannon's entropy or to compression algorithms, and they are mainly addressed to the comparison of strings of different length in inhomogeneous frameworks, a procedure motivated by the fact that in evolving sequences, beside substitutions, there are frequent insertions and deletions [15].

There are few remarkable alternatives to these “sequence based” type distances. Hemagglutination inhibition (HI) assays [16], reporting the ability of ferret antibodies, raised against one viral strain, to inhibit a second strain's ability to agglutinate red blood cells, are currently used to define similarity between antigens [17]. Certainly, the metrics extracted from HI tests is directly related to the real antigenic similarity between strains, but it requires HI assay animal data, which are difficult to obtain with high precision.

We intend to introduce an enhanced version of a different metrics, known as *Rohlin distance* [18], [19], which is based on the sequences symbols and is expected to be sensitive to their global distributions and correlations. It is also founded on the Shannon's entropy but, differently from other informational functionals, applies in a biologically homogeneous framework. Moreover it does not deal with the frequency probabilities of symbols on single sites, which are too poor as units and do not touch the global structure (see however [11] for interesting improvements in this directions). In our approach, the basic entities are indeed the partitions of a sequence into subsequences, as they are determined, starting from a configuration (the list of amino acids), by a projection operation: precisely, we consider the *partitions defined by homogeneous segments*. This aims at evidencing the ordered collection of connected subsequences of equal symbols. For instance, the alphabetical string 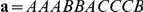 would be divided into five subsequences, and each subsequence would determine a segment. To the first segment 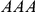 there correspond the site subset with labels 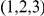, to 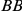 the subset 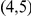, etc. The natural length of each segment is the number of its symbols. In the example, the lengths are 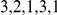. Once such lengths are correctly normalized, this assignment is equivalent to the definition of a probability measure on the subset algebra, proportional to the number of sites contained in each subset. Finally, partitions can be represented by their bounds, the segment extremes, or more economically by the left extremes, allowing for a simple and straightforward comparison between partitions.

In the partition space the Rohlin distance 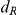 is then defined, for any couple of partitions 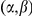, by the mutual conditional Shannon entropy (see Materials and Methods for details):
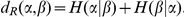
The conditional Shannon entropy 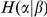 represents the residual information needed to describe the segment disposition of 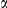 when the disposition of 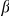 is known, or, in other terms, how the knowledge of 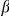 may contribute to the knowledge of 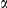. Therefore the symmetrized form above, defining 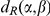, is the total information required to distinguish 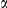 and 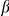, seen as schemes of segments with their probabilities.

In absence of a bias, our choice, assigning equal weight to each site and leading to a measure proportional to the length, is the most natural. Other probability measures can be defined, but they cannot depend on the configurations, since in that case the same set would have different measures for two configurations, and the conditional Shannon entropy would loose its meaning, inhibiting the very definition of Rohlin distance.

The segmentation provided by the partitioning of the sequences into homogeneous subsequences entails many advantages: its definition is simple and universal; sequences are not too tightly fractioned, as by single symbols; no *a priori* knowledge is required, along with the exigence of a “black box” analysis. Moreover, even if segments have no intrinsic biological meaning (and this could appear as an inconvenience), alterations in their overall distributions, as those emerging in historical records, are by definition compatible with the biologically efficient features proposed by evolution [20].

Thus, by using probabilities which arise from this geometrical and topological asset, the distance 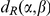 measures the information content carried by evolution, giving evidence to the emerging dissimilarities. Clearly, this content should be filtered, as far as possible, from the effects of the non evolving part: to this end we introduce the so called *reduction process* 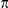, a method designed to amplify the relevant differences between partitions by dropping their common sub-partitions. This means that for any couple 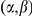 there is a reduced couple 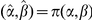 at amplified distance. From a practical point of view, the reduction consists in erasing the common extremes of segments between two partitions. A key point is that this method proves surprisingly effective also in filtering the noise of useless mutations. Details are given in the Materials and Methods section.

We shall deal here with an interesting example of a highly mutating sequence, the RNA of influenza A virus, whose databases are particularly rich. More precisely, we consider the amino acids sequence of the surface protein hemagglutinin for H3N2 subtype Influenza A virus [21], with human as host, in 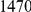 strains collected in USA from 1993/94 to 2010/2011 [22], and the analogous sequence for H1N1 subtype for 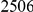 strains collected in USA from 2006/07 to 2010/2011. As our method works on equal length strings and it is based on an informational metrics with long range correlations, it is expected to perform better when applied to the longest sequences. We therefore choose the full length (566) HA sequences, which are a subsample of the available sequences. We also consider sequences identified by a complete date, as the time when the sequence appears represents an important information in our analysis. The restriction to the USA sequences is motivated by several facts. First, choosing sequences from the temperate regions, we give relevance to the seasonal timing of the virus evolution, minimizing the interference with a dephased development; second, the geographical bounds in the sampling ensure that we are looking, season by season, to a reasonably stable population. Notwithstanding these restrictions, for example for H3N2 we process 1470 sequences over 1986 in the northern hemisphere (more than 74 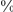) and over 2759 in the world (more than 53 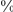). In other words, we keep the statistical majority, disregarding only possible noise. Analogous estimates hold for the processed H1N1 sequences.

After partitioning each sequence, we calculate the Rohlin distance between all the partition pairs, and analyze the whole sequences sample by the hierarchical complete linkage clustering algorithm [23] on the distance matrix. The procedure has strong analogies with the analysis presented in [9], [24] but in a completely different metric space, namely the partition space instead of the configuration space. Our analysis traces the evolution of topological properties of the sequences, while the virus escapes following its antigenic drift. Interestingly, the structures arising in the sequence space according to this metrics result to be quite meaningful; they individuate indeed well defined regions of the sequences space acting as *weak attractors* [9], [17], where the evolution of the virus takes place for definite periods. Moreover, the attractors display precursors [9], [17], i.e. sequences populating the regions well before they are identified as circulating strains, an information which appears to be relevant in the forecast problem, suggesting an alternative strategy in vaccines formulation. Once again there are analogies but also differences with the privileged regions of evolution presented in previous analysis [9]. Our attractors arise indeed from of similitude related to the overall disposition of homogeneous segments, and not to the actual data lying on every site (with the supplementary restriction to epitopes). This means that, more than an overlap of results, the two approaches present complementary points of view, enforcing one another. It is therefore quite remarkable that the vaccine predictions of the two methods agree very well, a fact whose origin is not completely understood yet.

## Results

### Clustering and weak attractors in Rohlin metrics

Using the sequences set, we calculate the whole 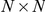 matrices of Hamming and Rohlin distances, i.e. 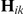, 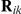 and reduced Rohlin distance 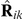, whose entries are respectively 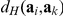 on sequences, or 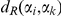 and 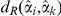 on the corresponding partitions (possibly simplified by the reduction process). The primitive (non-reduced) partitions result to be by far less informative than the reduced ones, so that from now on we shall omit to report calculations on them.

This fact is a non trivial result in itself: in principle, by deleting the common bounds, the reduction process could imply indeed the survival of a random set of unstable bounds created by the incoming mutations. This happens, as expected, with artificially created random mutations (as reported in the last subsection). Clearly, far from being more informative, such chaotic residual set would be useless for our scope.

The empirical fact we observe is quite different: once the reduction process has cleaned the useless common sub-partitions, the surviving bounds (defining the new reduced partitions), far from being “random”, carry the winning novelty in the adaptive strategy of the virus. In other terms, the effective new disposition of segments is not random because it has been selected. Moreover, the mutual disposition has intrinsically a long range character, which is captured by such a non local metrics as the Rohlin distance is (details on the amplification are given in the mathematical section in Materials and Methods).

**Figure 1:**
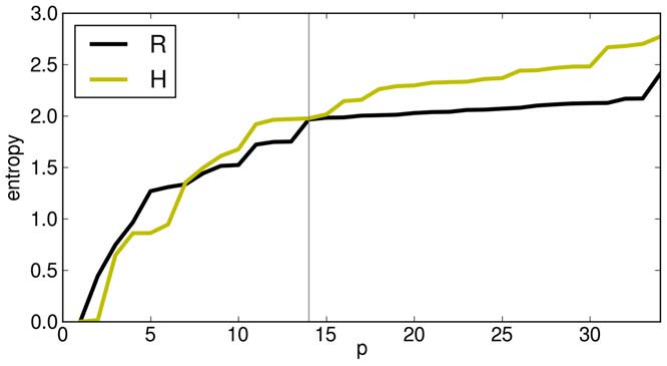
Looking for optimal 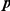
 **in clustering.** Clustering entropy for Hamming and Rohlin distance at different 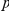 values for H3N2. The plateau, in Rohlin, suggests an optimal and stable result for the clustering.

The scenario emerges from a “clustering analysis” on the matrix 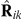. The used clustering tool is the standard hierarchical complete linkage algorithm [23], where the number 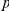 of clusters is an external parameter. An important point is therefore the choice of the optimal 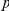. Since at fixed 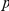 every clusterization corresponds to a partition of the whole set into groups populated by 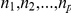 sequences, with 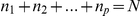, by defining the probabilities 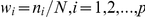 to each clusterization we can associate the Shannon entropy 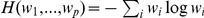 of its probability distribution. Since at growing 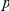 the cluster populations cannot increase, the entropy is non-decreasing [25]. However, if it is substantially stable, this means that the clusters are also stable (or equivalently, that newly added probabilities are very small); if it grows, then clusters (and probabilities) are almost continuously splitted into smaller and smaller ones. Now, observing the behaviors of Rohlin and Hamming entropies in Figure 1 for H3N2, we note a clear quasi-plateau for Rohlin in the interval 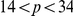, while Hamming is always growing. For the latter, the interpretation is that the Hamming clusters, being an artificial product of the procedure, split with a remarkable continuity.

As to Rohlin, the long plateau clearly indicates that the clusters are real structures in the sequences space, keeping a definite individuality in a large observation range. The growth for low 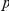 is simply due to the fact that, if the imposed number is too small, (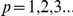), the calculated clusters must contain the real ones, so that at growing 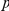 the splitting is effective, up to the optimal number when calculated and real clusters coincide. On the contrary, for 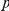 too large, calculated clusters are so numerous that also the real ones begin to split much more effectively than during the peripheral loss registered in the plateau. The plateau extremes may be therefore roughly related to the typical isolation length among real clusters, and to their maximal diameter respectively. Interestingly, the optimal value 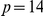, obtained from clustering without additional hypotheses, is consistent with the number of different circulating strains identified by the WHO HI tests.

Analogous results obtained for H1N1 are shown in Figure S1. In that case, the optimal value for the clustering parameter is 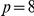.

**Figure 2:**
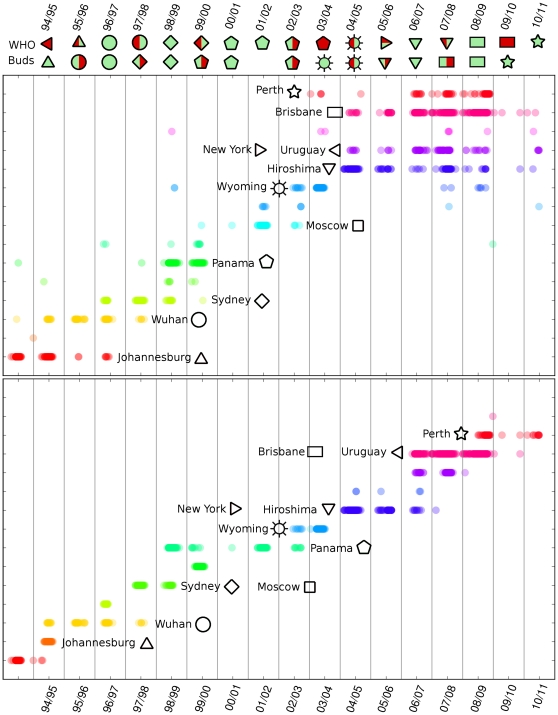
Clusters and time evolution for H3N2. (upper part) Rohlin clusters time evolution for H3N2. (lower part) Hamming clusters time evolution for H3N2. The reference WHO sequences are shown by the corresponding symbols and names, and the details are indicated in Table 1. In the upper part, we indicate the vaccine choice according to the WHO indication (up) and to buds criterion (lower). Green and red colors indicate right and wrong choice with respect to the corresponding analysis on the real circulating strain. A double color is used when more than one strain circulated in that year and the corresponding prediction agrees with one of the circulating strain.

Since every sequence is marked by its sampling date, a natural question is the time distribution of the resulting clusters. The upshots for H3N2 are summarized in Figure 2. The part above refers to the clustering on 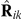. Below, for comparison, to the clustering on 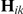. There is no scale on the 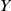 axis because the ordinate only distinguishes among clusters (same ordinate 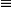 same cluster): order and color have no intrinsic meaning and they have been chosen with readability criteria only. The 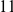 different polygonal symbols represent the 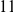 reference viruses, including the alternates, observed in the Northern Hemisphere according to WHO HI tests. Their names are indicated in the plot and the details of the sequences are given in Table 1. The reference sequences are not identified by a time 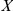 coordinate. They are processed together with the dataset and they are positioned in the clusters they belong to after the Rohlin clustering procedure. Vertical lines separate winter seasons and are conventionally set at July 31.

**Table 1:** Symbols legend for Fig. 2.

△	A/Johannesburg/33/1994	AY661180
◯	A/Wuhan/359/1995	AY661190
◊	A/Sydney/5/1997	EF566075
<$>\raster="rg1"<$>	A/Panama/2007/1999	DQ508865
□	A/Moscow/10/1999	DQ487341
<$>\raster="rg2"<$>	A/Wyoming/03/2003	CY034108
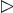	A/New York/55/2004	CY033638
▽	A/Hiroshima/52/2005	EU283414
▭	A/Brisbane/10/2007	CY035022
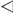	A/Uruguay/716/2007	EU716426
⋆	A/Perth/16/2009	GQ293081

**Table 2:** Symbols legend for Fig. 3.

◯	A/New Caledonia/20/1999	CY033622
▭	A/Solomon Islands/3/2006	EU124177
◊	A/Brisbane/59/2007	CY058487
⋆	A/California/07/2009	FJ969540

From the 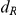 clustering of Figure 2, which is very stable by changing 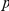 in the plateau range of Figure 1, we can draw several indications. First, there is a clear long temporal extension of clusters, which are densely populated for several winter seasons. Interestingly, they present precursors, that we term “buds”, and successors, i.e. a bunch of sequences representing viruses that appear, in time, before or after the main part of the cluster [9]. The identification of buds will be explained in details in the next sections.

Let us consider, for instance, the 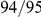 season. In that year, we observe a bud (Wuhan strain, the yellow cluster) which is getting stronger in the following season, living jointly with the Johannesburg strain, the red cluster. Then, it becomes the dominant strain in 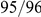 and 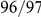, while it may be considered a successor in 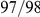, when the Sidney (light green) is the dominant strain. Notice that the same Sidney was a bud in 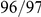.

The distribution of clusters suggests that the evolution in the sequence space takes place in preferential regions, corresponding to each cluster, which can be populated well before and after the main season. Such regions act as a kind of weak (i.e. non definitive) attractors. For example, as mentioned, two H3N2 virus strains circulated during 96/97 winter season: Wuhan successors and Sydney buds. Through HI tests, WHO revealed the Wuhan reference virus as the circulating one, recommending it as a vaccine for the season 97/98. It is crucial that, already during 96/97, our analysis shows the emergence of a bud, the Sydney family strain, which is the actual virus circulated in 97/98 winter season. Rohlin attractors correctly describe also the heterogeneity coming from “outliers” sequenced by the WHO, which must not be treated separately, as it happened in other Hamming based approaches [24]. These sequences naturally fall into a cluster, confirming that Rohlin correctly takes into account the variability present in outliers.

A second point is that 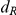-clustering is consistent with epidemiological WHO-HI data. For example, the subsequent strains A/Wuhan/359/95, A/Sydney/5/97 and A/Moscow/10/99, appeared during years from 95/96 to 02/03 according HI tests, are represented by three well defined clusters (A/Panama/2007/1999 is a Moscow alternate). Interestingly, their reference sequences belong to the correct clusters once they are included in the data set, without any a priori information.

In the lower part of Figure 2 the same clustering procedure is shown referring to the Hamming matrix 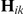. A definite temporal extension of clusters is observed, in agreement with previous results [9], [24]. However, the cluster temporal distribution obtained from 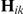 is quite unstable, confirming the dependence on 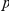 evidenced in Figure 1. The 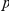 used here is the same of Rohlin, but the choice is completely arbitrary because there is not a clear plateau for Hamming. This means that some appearing spots are not true buds, as they results from the almost continuous splitting of Hamming clusters, which are not stable under a change in 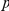. Namely, a new cluster can be produced simply by raising 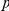. Moreover in some seasons (e.g. from 99/00 to 02/03) there is a contrast between WHO indication and the cluster arrangement: some clusters are not represented by any reference sequence, while others are wrongly doubly represented, showing a poorer correlation with HI analysis. For example, Sydney and Moscow reference strains belong to the same cluster while they are expected to be in different ones.

### Buds in Rohlin weak attractors and vaccine forecast

An interesting evidence can be drawn from the position of symbols in the attractors. The symbols above the upper diagram in Figure 2 show in the first row the vaccine indicated by the WHO on the basis of the HI tests in previous seasons, while in the second row they represent the indication we would suggest on the basis of the following criterion: by looking at the 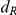-cluster distribution, when in a year there are simultaneous strains (with statistically significant populations), we would indicate the newest one, i.e. the bud, as emergent in the next year. This criterion, in other words, sees the novelty carried in emergent buds as a feature enhancing the aggressiveness of the virus. In both the WHO or buds analysis, the symbols are shown in green when the vaccine choice agrees with the circulating strain, in red when they disagree.We used a two-colors symbol when more than one virus circulated in the same season and the corresponding prediction agrees with one of the two circulating strain. Now, the second symbols row indicates that the buds criterion is able to identify correctly the circulating strain every year, apart from the season 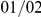 because of the lack of sequences, while the WHO criterion fails in 3 cases over 17. Of course, it will be extremely interesting to verify the criterion on the set of sequences for next season, as soon as they will be available.

In the lower part of Figure 2, displaying the results of the same procedure on the Hamming distance, we note that buds as early warning of new strains are not reliable because, as discussed above, the instability in 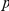 does not allow for an unambiguous detection of their appearance, as they can be produced by raising the value of 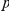.

**Figure 3:**
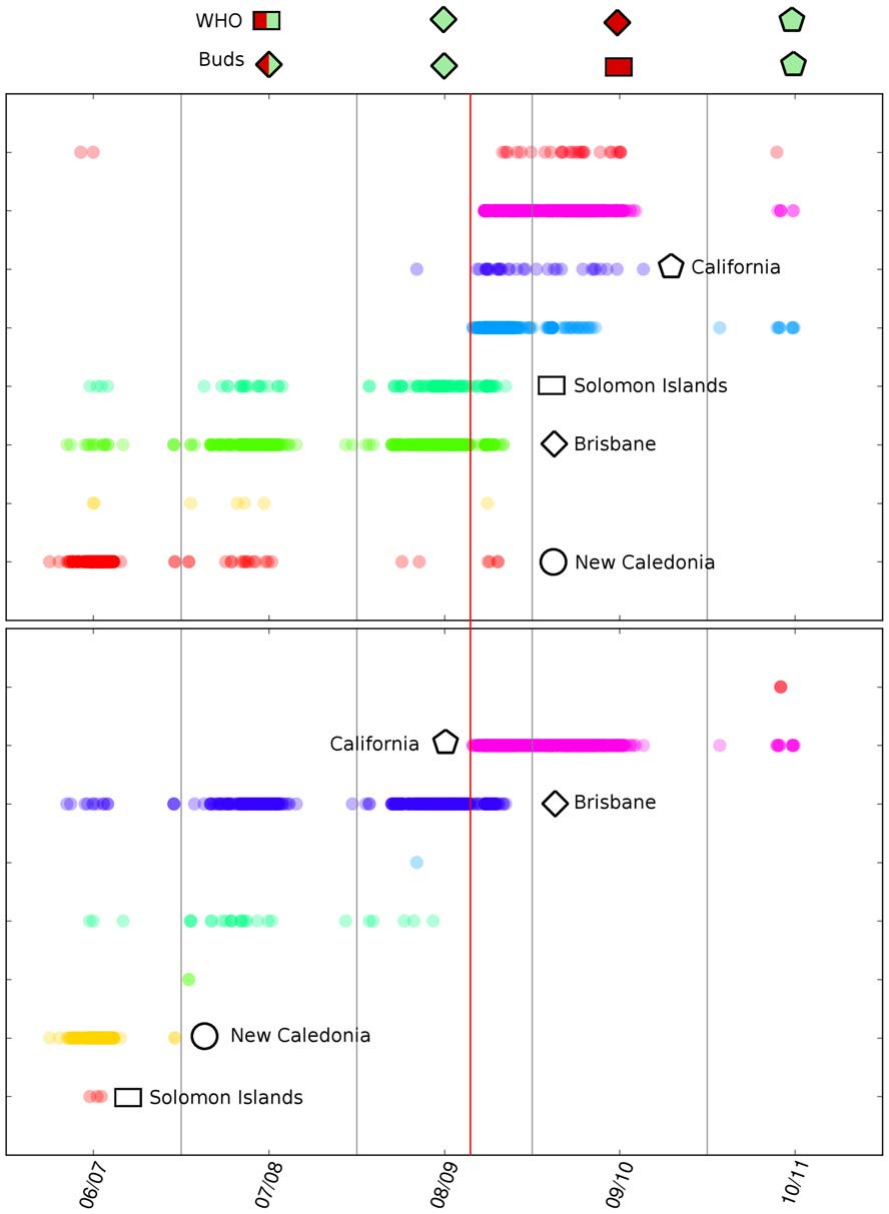
Clusters and time evolution for H1N1. (upper part) Rohlin clusters time evolution for H1N1. (lower part) Hamming clusters time evolution for H1N1. The reference WHO sequences are shown by the corresponding symbols, as indicated in Table 2. In the upper part, we indicate the vaccine choice according to the WHO indication (up) and to buds criterion (lower). Green and red colors indicate right and wrong choice. In this case, some of the symbols does not correspond to a specific HI test, so they are indicated by a star and a pentagon. Notice the onset of the pandemic virus and the failure of the bud criterion after that line.

The bud criterion can be successfully applied also to the very interesting case of H1N1 and the results are shown in Figure 3. The sampling for H1N1 is very inhomogeneous. From 2006 the statistics increases, so we will limit our analysis to this period. From the clustering entropy analysis (shown in Figure S1), the optimal 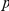 is 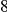. The very relevant cluster starting suddenly around April 2009 (red line in Figure 3) [26] represents precisely the pandemic virus appeared in the 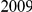 season. In that case, the bud criterion partially fails, as it recognizes correctly only the strains circulated at the beginning of the season. This is reasonable, since our method is expected to be effective in simple antigenic drift, and not in the case of a dramatic change, as in the 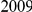 pandemic case. The shift probably sets a completely new “direction” in the sequence space. Notice that our method evidences the simultaneous occurrence of four well distinct clusters in 2009, a feature missing in the Hamming analysis. The observation of multiple clusters signals could be related to a typical instability of post-pandemic periods, as the one we are facing according to the WHO [26]. We expect this structure to be present also in the new set of sequences for season 2011/12.

### Restricting the time window

**Figure 4:**
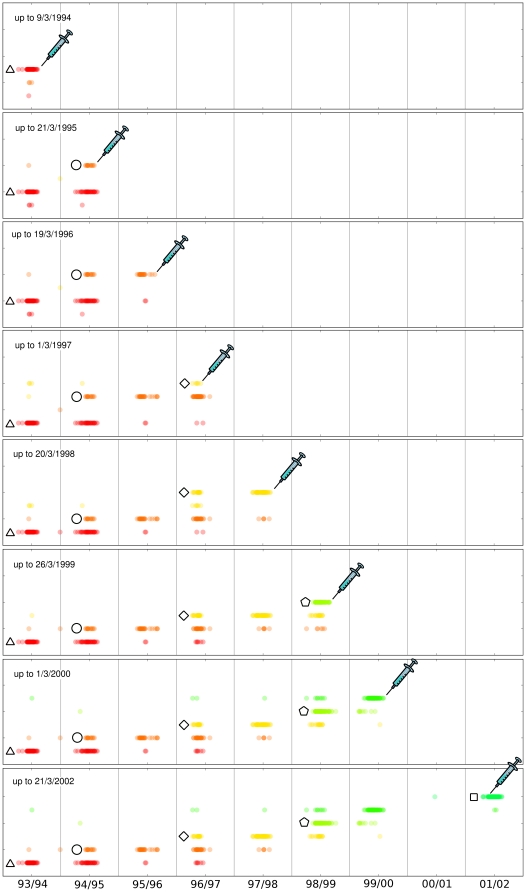
Changing the time window. Rohlin analysis during years. Clusters structure is robust under a sampling increase, and buds appeared during seasons correctly reveal the future circulating strains, as indicated by the syringe.

Another natural question, the relevance of the examined time window, is treated in Figure 4, displaying the results one would obtain by stopping the data collection at five different years, i.e by applying the clustering to the restricted sets of sequences available at those times. This procedure is intended to clarify how the bud criterion works and to check that it is not an unpredictive *a posteriori* verification of the vaccine choice, but a real working framework.

The time distribution in the 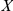 axis describes at various years the position of the H3N2 sequences, exactly as in the upper Figure 2. However, to reproduce exactly the situation in which the WHO vaccine prediction is made, we collect all the sequences available up to March of a given year and we perform our clustering analysis only on that dataset. There is therefore an increasing number of sampled sequences, in every horizontal time sector starting for the upper part down to the lowest. In details, we apply our entropy criterion each time to the dataset restricted to the end of the winter season of a given year and, from that dataset only, we choose the optimal 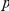 for that case. Interestingly, there is a clear plateau in the entropy analysis for every restricted time window, allowing for a unambiguous choice of the number of clusters. The Rohlin entropy analysis from the restricted time window is shown in Figure S2, together with the corresponding one for Hamming. Notice that for the Hamming clustering, there is no clear indication of the number of clusters 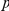 from the entropy analysis, nor any evidence of emergent buds.

In principle, leaving out part of the data, these clusterings could be different from the final one. It is remarkable that the structure remains the same. Buds are clearly present, as if the evolution took place in a well defined landscape, with preferential “antigenic” directions that are filled during the genetic drift, and acting therefore as weak attractors. The symbols of the reference WHO strains are excluded from the clustering in the time restricted window, as they would represent an a posteriori knowledge. They are associated to each cluster by an inverse analysis, i.e. by calculating the reference WHO strain which has minimum distance with the sequences belonging to the bud cluster. Details on the reverse analysis, applied also to the whole dataset are given in the next subsection. Now, in Figure 4 a syringe indicates the most accredited vaccine for the next year based on the bud criterion: even when the previous years database is poor, the forecast is very good and the cluster corresponding to the syringe is exactly the prevailing cluster observed the next year. By confirming the coherence of the procedure, this result supports the bud emergence criterion for the prediction of the new prevailing strain.

Interestingly, our bud criterion, which does not include any additional information on epitopes positions, agrees very well with the dominant strain prediction discussed in [9]. This appears to indicates that the “black box” Rohlin distance analysis is able to grasp the biological information included in the “epitopes” metrics, which is certainly correct but requires an additional input (the relevant positions). We notice that our results for the vaccine choice always agree with the prediction of [9] when there is a single circulating strain, while they are complementary when there are two. We do not have a clear explanation for this interesting fact, at the moment.

### Testing the method: reverse analysis and random permutations

The clustering procedure can be performed by a completely different method, which does not consider distances between sequences themselves, as in the hierarchical method, but refers to the WHO different sequences identified by the HI analysis. Precisely, the Rohlin distance has been calculated between each of the 1470 sequences of the H3N2 with all the 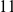 WHO reference virus strains. The sequences are temporally aligned along the 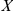 axis of Figure S3, while the reference strains have a conventional position along the 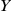 axis. In the diagram, each sequence is represented by a point whose 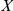 coordinate is its sampling date, and whose 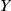 coordinate corresponds to the *nearest* WHO reference strain. Surprisingly, the final result of this new procedure is almost the same as the one showed in Figure 2 of the main text, while it is obtained from a completely different analysis. The analogous plot on Hamming distance does not preserve the cluster structure. Once again, the Rohlin distance approach proves robust and consistent with the HI tests analysis.

As a further check of the robustness of our results, we consider a random permutation of the site labels, simultaneously performed on all the sequences of the H3N2 dataset. This operation leaves the Hamming distances invariant by definition. Since such a random mixing of the amino acids is biologically meaningless, a natural request is that the conclusions drawn from a correct metrics should crash: this is precisely what happens with the Rohlin distance. In other words, with Rohlin, only the partitions corresponding to the real sequences seem to encode correctly the antigenic drift during the evolution, evidencing a meaningful relation with the global structure of the sequences. Vice versa, the simple global “mutations counting” completely fails to recognize the information deletion caused by the label permutation. The results are presented in Figure S4.

## Discussion

The mechanism underlying influenza A antigenic plasticity, that is, how the virus continually escapes the immune system by producing variant strains that cause re-infection within a few years, remains an outstanding evolutionary problem.

There are two main general pictures for this evolution. The first one is based on an almost continuous slow drift from an ancestor sequence, with some large shifts occurring at certain stages of the evolution [27]. The second one relies on a punctuated evolution, where “antigenic” swarms of sequences populate “basins” in the sequence space for several years, until the circulating swarm “jumps” to another basin, reinfecting the population. Dynamical model for this type of evolution have been built [28], and the evidence of a punctuated antigenic evolution has been put forward by several authors [17], [21], [29]–[31].

The picture emerging from the Rohlin metrics seems to support such a punctuated evolution with a better fit of epidemiological data, giving also insights on the relevant distance between circulating strains and vaccines. In fact, clusters result to be organized into well defined regions of the sequences space: the virus appears to explore for several seasons the sequence space region corresponding to a certain Rohlin width, until a jump takes it to another attractor, where the evolution starts again (a return to a previous region is also possible). These regions constitute therefore *weak attractors* in the sense that they are able to trap the virus for a finite time, and they can also be re-populated after years. In other words, weak attractors seem to identify privileged antigenic directions from genetic data.

Interestingly, the clusters present “precursors”, that we termed *buds*, i.e. a small number of sequences which explore in advance the next attractor when most of the strains still belong to the previous one. All goes as if such precursors manage to experience the winning escape strategy, that will be followed by the main swarm in subsequent years, and a clear correlation emerges between the bud, i.e. the younger attractor appeared in a given year, and the circulating strain of the subsequent season. This *bud criterion*, in parallel with HI analysis, could be helpful in the correct choice of the vaccines. The picture emerging from Rohlin distance analysis appears to hold also by processing analogous data sets as the A/H1N1 in USA. Interestingly, in H1N1 the bud criterion partially fails in 2009, as it recognizes correctly the emerging bud only before the pandemic period, while it is not able to predict the clear new cluster that appears suddenly in April 2009. The analysis correctly signals also the high instability of the post-pandemic phase in 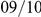.

In conclusion, some main points should be stressed: the first is that no *a priori* knowledge of biological nature has been used or put into the data set. The indications we have derived from clustering on the distance matrix constitute a genuine emergence. It seems plausible therefore that the same approach could work in similar circumstances, i.e. when a homogeneous set of equal length arrays are at disposal. The second point is the existence of structures in the sequence space, that can be described as weak attractors, where the evolution of the viral species takes place with a discontinuous dynamics. Clearly, a clustering algorithm is expected to recognize a chronological order within the distance matrix, whenever the distance is a monotone function of time, but in that case one would also expect the progressive fragmentation of clusters as the external parameter 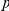 grows. Such is, substantially, the behavior suggested by the analysis on the Hamming matrix. On the contrary, the presence of precursors [9], which discontinuously anticipate the onset of future attractors, and the stability of the attractors structure at varying 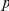 or sampling, are quite non trivial facts, implying that the Rohlin attractors are not a conventional decomposition in the sequence space; they possess instead a robust, intrinsic, “natural” meaning. It seems therefore that the Rohlin distance on reduced couples is able to evidence a selected variety of admissible “antigenic states”, preferentially explored through mutations, which remains hidden in other metric approaches. The third point is that the “buds emergence criterion” could offer a valuable complementary tool for an optimal strategy in the choice of vaccines. The matter is obviously delicate, and a long series of experimental checks should explore and confirm such a possibility before practical utilization, but we think that an effort in this direction is worthwhile.

## Materials and Methods

### Data

The main database of reference for H3N2 is constituted by 1470 full-length (566 aa) HA proteins of the H3N2 subtype Influenza A virus isolated in USA from 1993/94 to 2010/11, excluding sequences with an incomplete sampling date. Such set is enriched with the 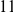 reference sequences corresponding to the reference viruses circulated in the same years according to WHO HI analysis [32]. The total is 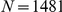 sequences, written in the 20 amino acids alphabet. As for H1N1, the main database of reference is constituted by 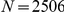 full-length (566 aa) HA proteins of the H1N1 subtype Influenza A virus isolated in USA from 2006/07 to 2010/2011, excluding sequences with an incomplete date. Such set is enriched with the 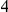 reference sequences, corresponding to the reference viruses circulated in the same years according to WHO HI analysis [32]. The total is 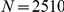 sequences, written in the 20 amino acids alphabet.

### Sequences and Rohlin Metrics

Let 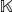 be a finite alphabet of characters (amino acids in our case). A sequence 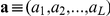, where 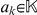, may be thought as a function on the one dimensional array 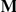 of sites labeled 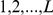. This function defines the *configuration* or *state* on 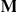. Every sequence 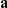 is therefore an element in a configuration space 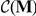. A probability measure 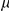 on the finite subset algebra 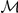 of 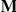 is given by the normalized number of sites in every subset. This means that all sites are assumed to be equivalent. For instance, if 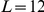 and a subset 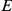 includes sites 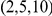, then 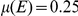. Other measures are possible, e.g. by assigning weights to the sites. However, such weights (and measures) cannot depend on the configurations, otherwise the same subset of 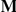 could have more than one measure simultaneously, and the functionals defined below would loose any meaning.

A partition 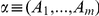 of 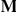 is an exhaustive collection of disjoint subsets (called “atoms”) of 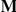. The space 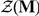 is the set of all possible partitions of 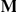, where a partial order 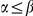 means that 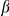 refines 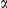. The “product” 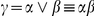 (a close analogous of the minimal common multiple) is the minimal partition refining both factors 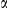 and 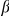. The unit partition 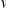 has only one atom, the whole set 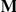. Obvious properties such as 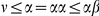 easily follow for every 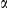 and 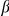.

The operation 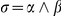 is the maximal common factor (i.e. the most refined common sub-partition) of 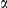 and 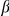. Clearly, 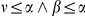, etc.

Every partition may be thought as an experiment where an elementary or *atomic* event 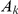 occurs with probability 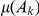. Then, the meaning of the definitions above is that 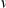 is the trivial experiment, 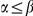 means that 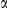 is a sub-experiment of 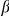, etc.

The Shannon's entropy 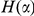 is defined as:
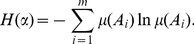
(1)If 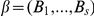, is another partition, the conditional entropy 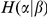 is
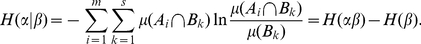
(2)These quantities give respectively the mean incertitude of an experiment 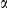 and the residual mean incertitude on 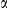 when the result of 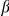 is known [25]. Now, for all 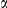 and 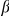 in 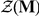, the Rohlin distance is:
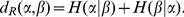
(3)The useful formula
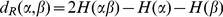
(4)follows from Eq. 2. Thus, 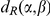 is a measure of the overall non-similarity between 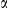 and 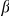, giving account of the mutual correlations among the respective outcomes [18], [19]. These concepts and definitions hold true in all probability spaces. For discrete spaces (graphs or lattices), where the *states* or *configurations* are determined by the values assumed by sites 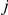
's in a finite alphabet 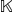, 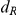 is therefore deeply different from the well known Hamming distance 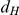 between configurations 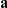 and 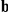. This distance is defined, up to a possible normalization factor, by:
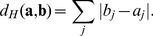
(5)where 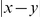 is a distance in 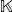 if the alphabet is numerical, otherwise is 1 for 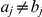 and 0 for 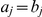 (as in our case, since 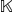 is the alphabet of amino acids). Counting the sites with different symbols regardless of their position, 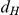 tells one nothing about correlations between mutations. It is important to stress that the Hamming and Rohlin distances are not defined on the same objects, the former being between configurations in 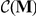, the latter between partitions in 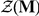.

In our particular case, where 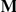 is a one-dimensional finite lattice, and the states (or configurations) are character sequences of length 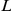, we shall work with partitions generated by *homogeneous segments*, i.e. consecutive sites with the same value in 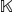. Of course, in 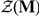 there exist much more partitions, e.g. those with non connected atoms. As an example with 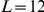, consider the fictional configuration 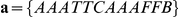 The atoms (indicated by the site labels) of the corresponding partition 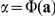 are 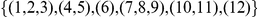. The map 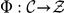 is univocal but non invertible, since several configurations are mapped into the same partition. For instance, a mutation from 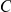 to 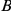 as in 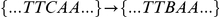, does not affect the boundaries, and it leaves the segment structure unchanged. Thus, by the correspondence 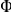 and the Rohlin distance, we can evaluate “how different” are the states on 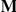 with regard to the correlated distribution of segments. It is true that there is a loss of information due to the projection of many configurations into the same partition; but a comparable loss takes place also for Hamming, since the single site contribute gives account only for the “equal-or-not” distinction in 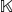. Moreover, as noticed, sites in 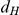 are always totally uncorrelated.

The non-similarity between two partitions could be confused and weakened by the presence of a tight common factor, that we would eliminate as far as possible, in order to amplify the Rohlin distance giving evidence to the real emerging novelty. However, such a “reduction” operation (analogous to the reduction to minimal terms for fractions) is not uniquely defined because partitions do not admit a unique factorization into primes [33], [34]. The role of prime (i.e. indecomposable) factors can be played by *dichotomic* sub-partitions, which are still extremely redundant. Then, the key point consists in defining for each partition a restricted family 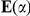 of “elementary” dichotomic factors, with the following features:

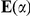
 must be well defined for every 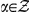, or at least in the subset of 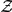 actually under investigation;

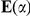
 does not contain more factors than the number 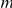 of atoms in 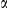;

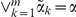
.

Now, assuming that the elementary factors families 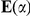 and 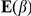 have been defined, the reduction process consists in the following steps:

1. define the maximal common divisor 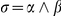;
2. drop from 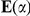 and 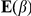 those factors which are not relatively prime with 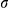, and note the surviving factors 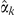 and 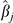 respectively (i.e. 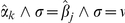);
3. define 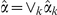 and 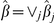.

In other words, we drop those dichotomic factors which are subfactors of the maximal common factor 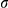, and the reduced 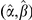 are generated by the surviving factors. The amplification of non-similarity is a consequence of the following property:

Proposition: 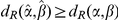.

The proof is elementary recalling that, if 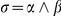, we can write 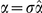 and 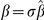: indeed, as mentioned, 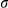 contains all the factors dropped during the reduction. Therefore, by formula (4) and the fact that 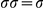, the thesis 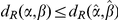 can be rephrased as
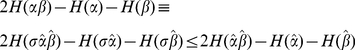
(6)Moving terms between the sides
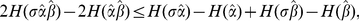
(7)and using formula (2) for the conditional entropy, the thesis reduces to
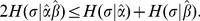
(8)But this is clearly true since
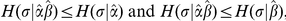
because the conditioning terms are greater in the left sides, q.e.d.

It is important to remark that this amplification regards the anti-similarity of the couple as a whole, while for reduced partitions as single entities the complexity possibly decreases, as expected: this means 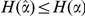, etc.

The correspondence 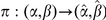 defining this reduction process is many-to-one and idempotent. It is a projection from 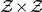 on the subset of irreducible pairs. The process, therefore, essentially depends on the family of elementary factors, a choice which *a priori* can be implemented in many ways, reflecting the kind of interest the observer has in the experiment. Details and procedures in abstract probability spaces may be found in [33], [34]. Here we sketch an algorithmically easy recipe, fitting the very special case of character strings.

By exploiting one-dimensionality, a partition into segments (connected subsequences) can be economically represented by the list of the left bounds of segments. In the example above, 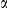 is fully determined by 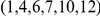.

**Figure 5:**
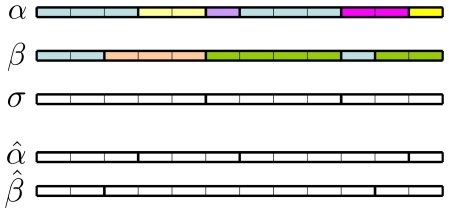
Reduction. Two partitions 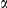 and 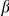 (derived from sequences as given in the text) and their maximal common factor 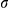. Thick vertical lines individuate the atomic segments. Below, the reduced partitions 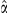 and 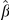. Colors in 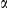 and 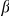 remind the source configurations. There are no colors in 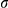, 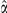 and 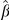 because these partitions do not originate from sequences of symbols but are directly defined in the partition space.

This suggest a very convenient choice of the family of elementary factors: precisely, for every 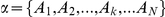, the 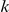-th dichotomic factor 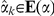 is
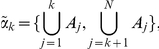
and therefore, in terms of labels, the example above for 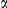 gives 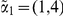, 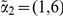, etc. With such a choice, the reduction process 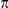 described above consists in erasing all the common labels apart the first one (label 1 is indeed the necessarily common bound for alignment). For instance, consider again 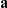 as above, and a new configuration 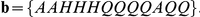 The list for 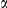 is 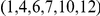, the list for 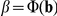 is 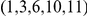, the list for 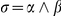 is 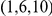. Then, the reduced 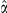 and 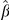 are represented by 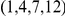 and 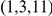 respectively. Note that they do not correspond to any new sequences, since the reduction is performed directly in 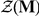, not in 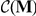. A graphic intuitive representation of this reduction is given in Figure 5.

## Supporting Information

Figure S1
**Looking for optimal** 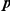 **in clustering for H1N1.** Clustering entropy for Rohlin and Hamming at different 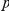 values for influenza A H1N1. The long plateau, in Rohlin, suggests a stable and well defined value for the optimal 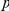. Notice that Hamming is growing.(TIF)

Figure S2
**Looking for optimal** 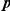 **in clustering for H3N2 in the restricted time window.** Clustering entropy for Rohlin and Hamming at different 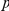 values for influenza A H3N2, as obtained by considering only the sequences up to the end of the winter season of the year indicated in the plot. In each time window, the long plateau, in Rohlin, suggests a stable and well defined value for the optimal 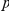. This figure is in correspondence with Fig. 4 of the main text.(TIF)

Figure S3 **Reverse analysis for Rohlin clusters.** Sequences of minimum distance with the corresponding WHO reference sequences, during years. The great similarity with Fig. 2 shows a strong consistency between Rohlin and HI analysis.
(TIF)

Figure S4
**Clustering on random permutations.** Effect of random permutation of symbols on the entropy of the clustering, as a function of 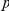. R indicates the entropy of clustering with the Rohlin distance and P stands for the entropy of clustering in the sample, obtained under a random permutation of symbols in each sequence.(TIF)
