# Correction: Rohlin Distance and the Evolution of Influenza A Virus: Weak Attractors and Precursors

**DOI:** 10.1371/annotation/bcc81b1a-608d-4814-b287-7a5ce3d7ee36

**Published:** 2012-01-11

**Authors:** Raffaella Burioni, Riccardo Scalco, Mario Casartelli

There is a spelling error in the funding. The correct funding statement is: This work was partially supported by the MIUR FIRB grant RBFR08EKEV and by the Istituto Nazionale di Fisica Nucleare (INFN) project “Biological applications of theoretical physics methods”. The funders had no role in study design, data collection and analysis, decision to publish, or preparation of the manuscript. 

