# Correction: Rohlin Distance and the Evolution of Influenza A Virus: Weak Attractors and Precursors

**DOI:** 10.1371/annotation/289e7480-aaa8-42b5-8a24-e45030f58dca

**Published:** 2012-01-11

**Authors:** Raffaella Burioni, Riccardo Scalco, Mario Casartelli

There is a spelling error in affiliation 1. The correct affiliation 1 is: Dipartimento di Fisica e Istituto Nazionale di Fisica Nucleare (INFN), Università di Parma, Parma, Italy

