# Supplementary figures and images for: Rohlin Distance and the Evolution of Influenza A Virus: Weak Attractors and Precursors
Source: PLoS One. 2011 Dec 6;6(12):e27924. doi: 10.1371/journal.pone.0027924 (PMC3232212; doi:10.1371/journal.pone.0027924)

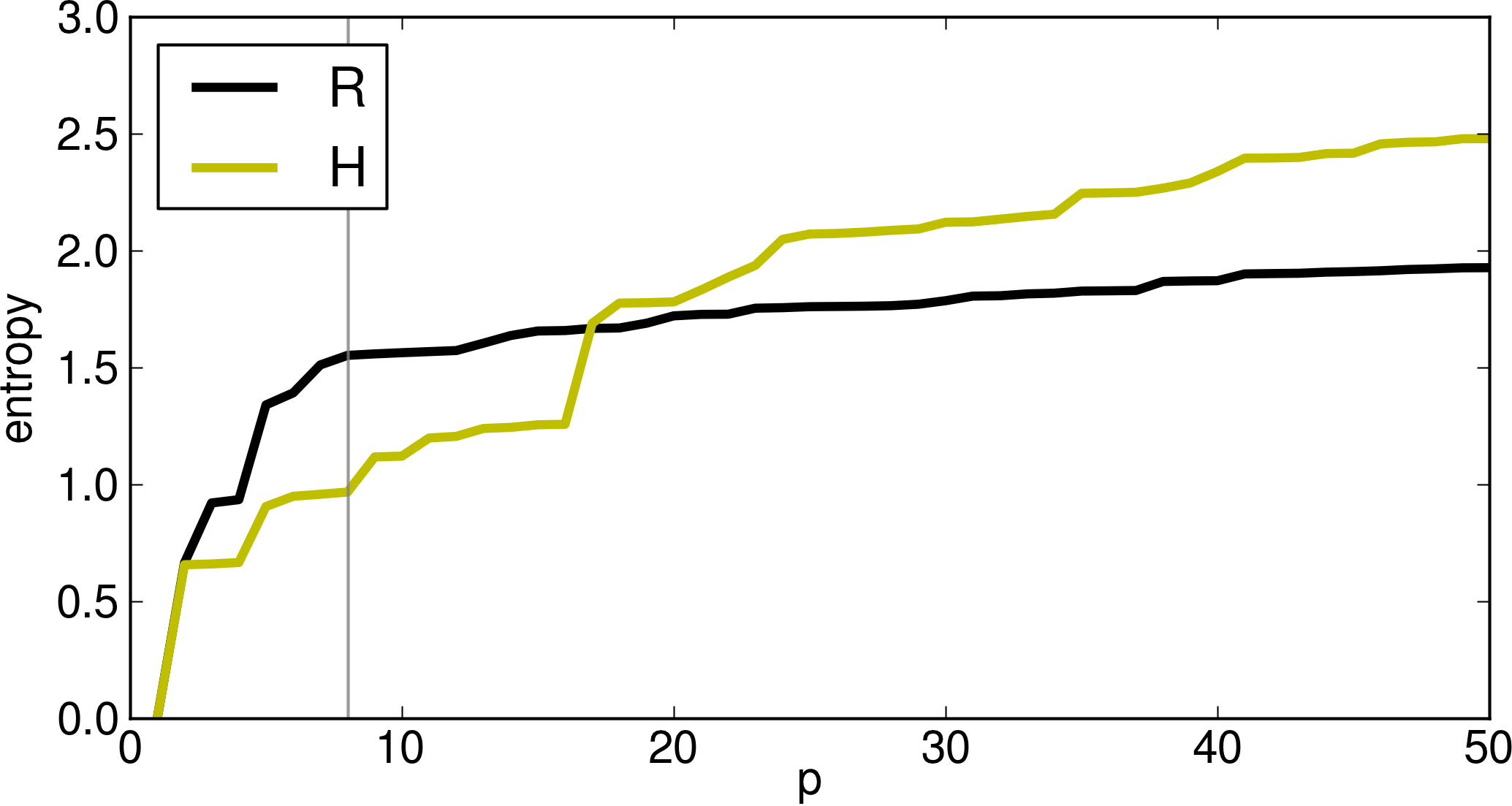

Supplement: Figure S1 — Looking for optimal in clustering for H1N1. Clustering entropy for Rohlin and Hamming at different values for influenza A H1N1. The long plateau, in Rohlin, suggests a stable and well defined value for the optimal . Notice that Hamming is growing. (TIF) [file pone.0027924.s001.tif]

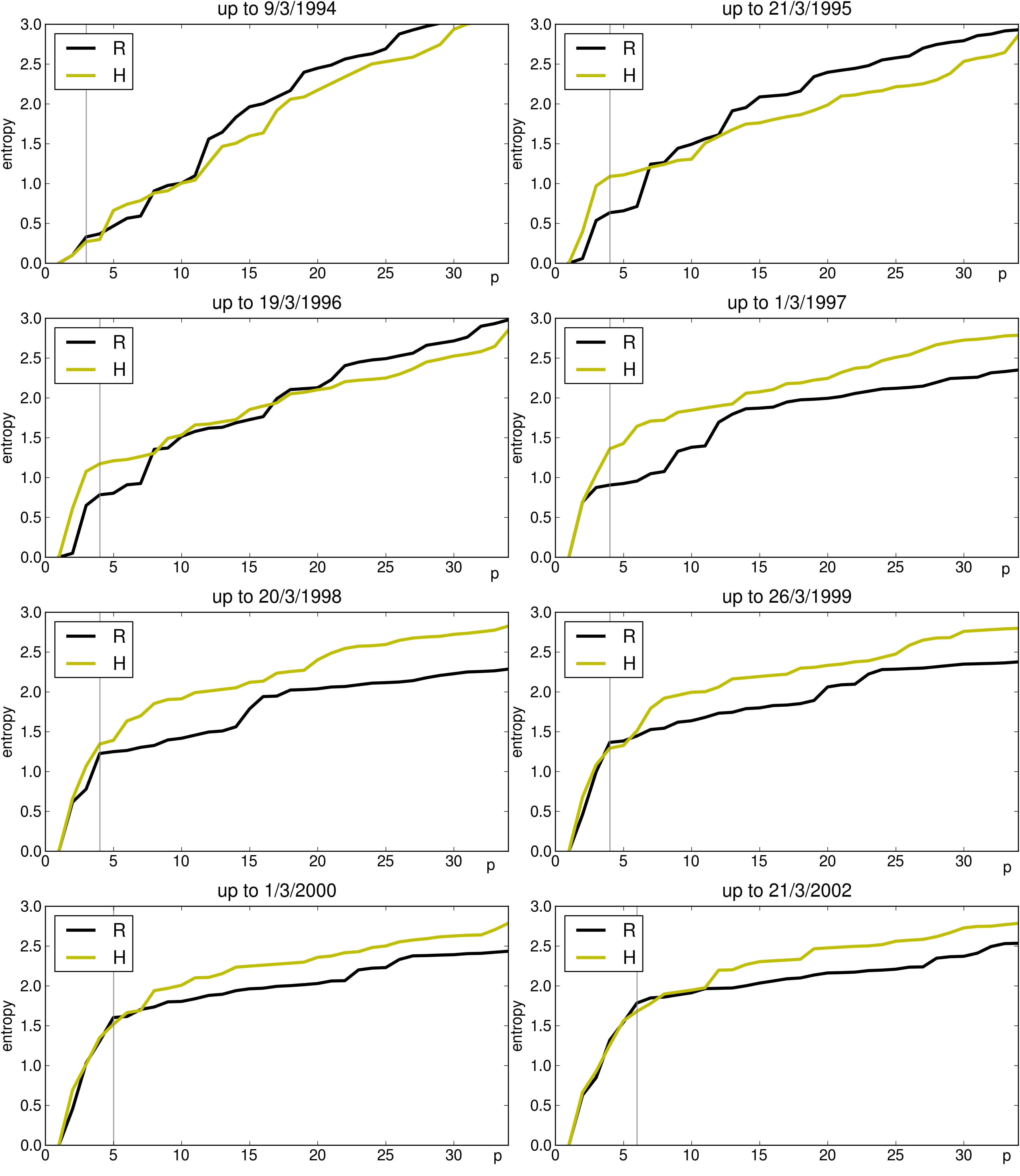

Supplement: Figure S2 — Looking for optimal in clustering for H3N2 in the restricted time window. Clustering entropy for Rohlin and Hamming at different values for influenza A H3N2, as obtained by considering only the sequences up to the end of the winter season of the year indicated in the plot. In each time window, the long plateau, in Rohlin, suggests a stable and well defined value for the optimal . This figure is in correspondence with Fig. 4 of the main text. (TIF) [file pone.0027924.s002.tif]

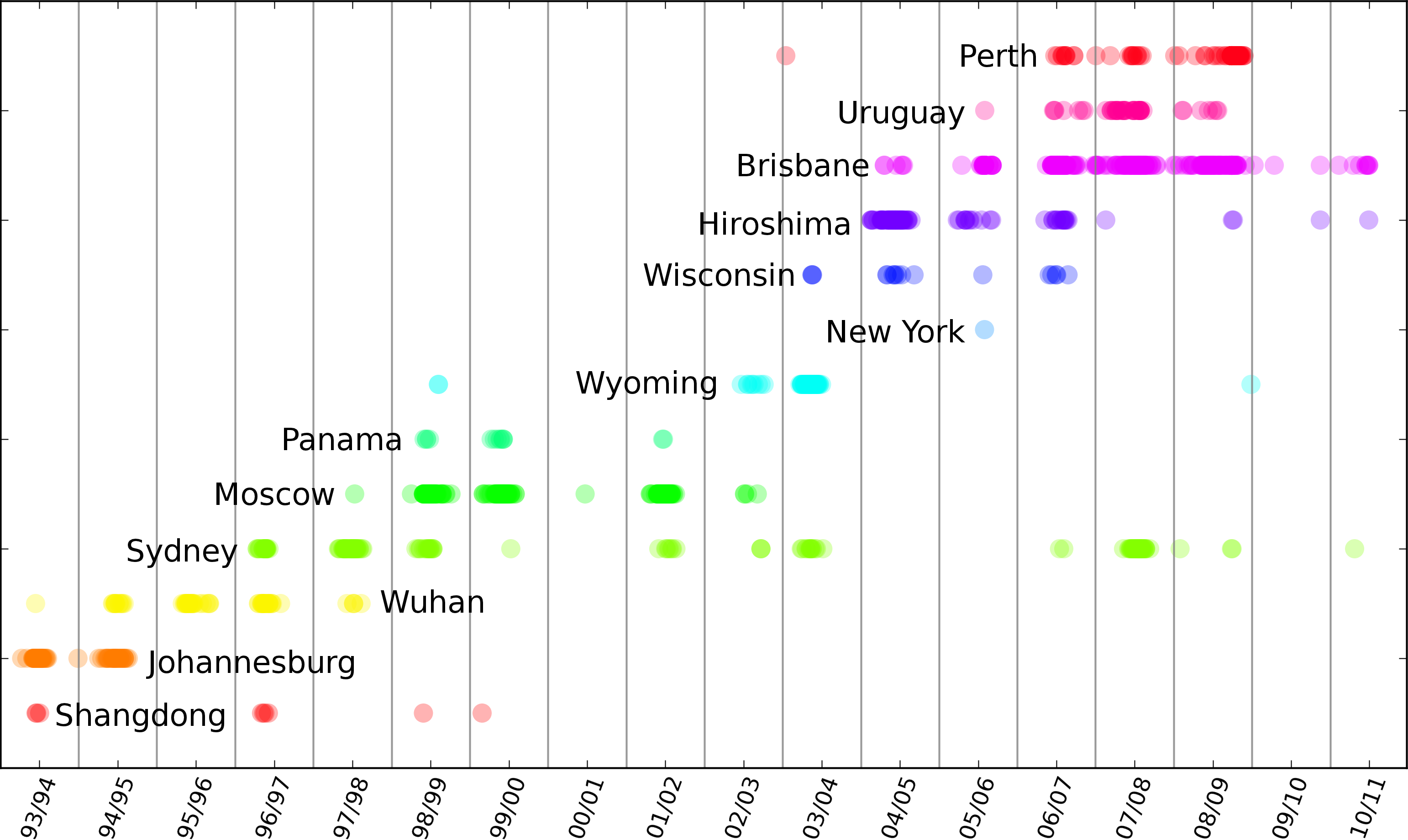

Supplement: Figure S3 — Reverse analysis for Rohlin clusters. Sequences of minimum distance with the corresponding WHO reference sequences, during years. The great similarity with Fig. 2 shows a strong consistency between Rohlin and HI analysis. (TIF) [file pone.0027924.s003.tif]

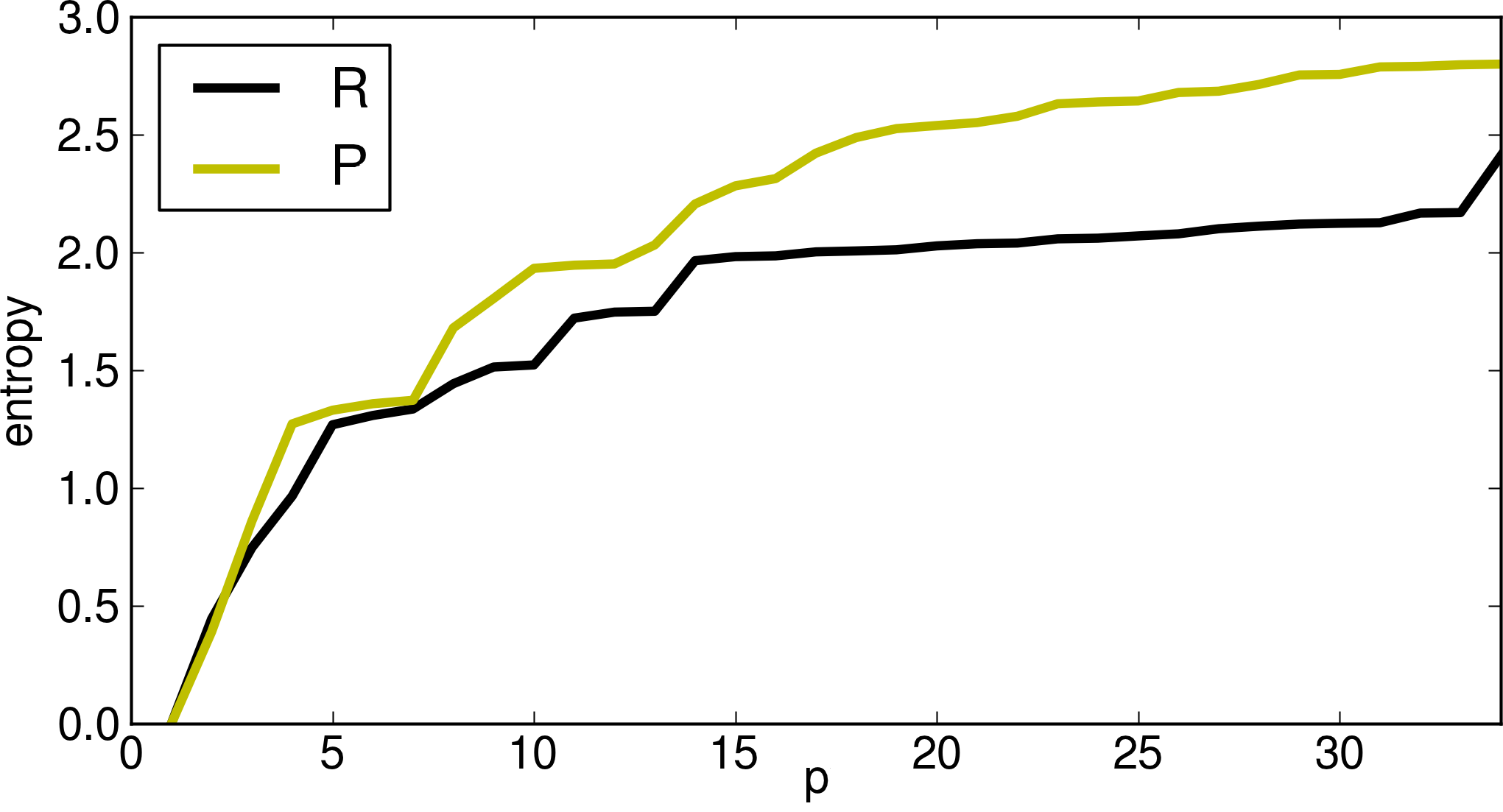

Supplement: Figure S4 — Clustering on random permutations. Effect of random permutation of symbols on the entropy of the clustering, as a function of . R indicates the entropy of clustering with the Rohlin distance and P stands for the entropy of clustering in the sample, obtained under a random permutation of symbols in each sequence. (TIF) [file pone.0027924.s004.tif]
